# Regression of primary cardiac angiosarcoma and metastatic nodules following propranolol as a single agent treatment

**DOI:** 10.18632/oncoscience.472

**Published:** 2018-10-11

**Authors:** Dana C. Galván, Anoop P. Ayyappan, Brad A. Bryan

**Affiliations:** ^1^ Paul L. Foster School of Medicine, Texas Tech University Health Sciences Center, El Paso, TX, USA; ^2^ Department of Radiology, Texas Tech University Health Sciences Center, El Paso, TX, USA; ^3^ Department of Biomedical Sciences, Texas Tech University Health Sciences Center, El Paso, TX, USA

**Keywords:** angiosarcoma, cardiac tumor, metastatic, propranolol, beta blocker

## Abstract

Angiosarcoma is the most common malignant cardiac tumor. Cardiac angiosarcoma is a highly lethal neoplasm that is largely resistant to conventional anti-cancer therapy. Mean survival of patients with cardiac angiosarcoma is only 4 months, and almost all patients will succumb to the disease within 1 year. The beta blocker propranolol is an emerging therapy against angiosarcoma. When combined with conventional therapies, propranolol increases progression free and overall survival in patients with this tumor type. It is currently unknown if propranolol is capable of showing anti-cancer efficacy as a single agent therapy. We report a case of a 61 year old woman diagnosed with primary cardiac angiosarcoma and liver and lung metastases. This patient chose to decline conventional therapy, and instead was prescribed the beta blocker propranolol as a single agent treatment. After 12 months, the mediastinal mass substantially debulked and decreased in size, and the metastatic nodules stabilized or resolved with no evidence of hyper-metabolic activity on PET-CT. This is the first reported data showing long term efficacy of the beta blocker propranolol as a single agent therapy against angiosarcoma.

## INTRODUCTION

Primary cardiac neoplasms are exceptionally rare tumors, with an autopsy incidence of 0.0001% to 0.03% [1]. The most common malignant cardiac tumor is angiosarcoma, an endothelial cell sarcoma that comprises approximately 2% of all primary cardiac neoplasms [2, 3]. Cardiac angiosarcomas predominantly occur in males who are less than 65 years of age [4, 5], and have been shown, at least in some cases, to display a familial pattern of inheritance corresponding to a germline POT1 mutation [6, 7]. Patient diagnosis is often delayed as a result of presenting vague, generalized symptoms that initially appear relatively benign including shortness of breath, weight loss, and fatigue associated with anemia [8]. A definitive diagnosis of angiosarcoma is generally confirmed using a combination of imaging, histology, and positive expression of vascular protein markers including CD31 and CD34 [9-13]. Metastatic disease is very common at the time of diagnosis, with most disseminated tumors occurring in the liver, lymph nodes, bone, adrenal glands, and spleen [14].

Nearly 80% of cardiac angiosarcomas arise in the right atrium where they typically replace the atrial wall, fill the entire cardiac chamber, and aggressively infiltrate into the surrounding pericardium. Extensive pericardial spread and encasement of the heart occurs rapidly, leading to congestive heart failure, pericardial effusion, and cardiac tamponade. Surgical excision of the mass is the preferred treatment option, however the rarity of cardiac angiosarcoma has made treatment difficult to standardize and the benefit of adjunctive chemotherapy and/or radiation is currently unknown. Even with comprehensive therapy, mean survival is only ~4 months, and greater than 90% of patients die within 1 year of diagnosis [14-18].

Emerging evidence from a number of retrospective clinical analyses and prospective case reports indicates that the beta blocker propranolol exhibits clinical efficacy against primary and metastatic angiosarcomas [19-26]. Due to a dearth of effective treatment options against angiosarcomas, and the remarkable clinical outcomes reported in the literature for propranolol, this beta blocker has recently received Orphan Drug Designation against angiosarcomas by the European Medicines Agency. Durable efficacy of propranolol against angiosarcomas has been reported when this drug is used in combination with conventional therapies such as surgery, chemotherapy, and/or radiation. To date, no studies have examined the long-term efficacy of propranolol as a single agent therapy against angiosarcoma.

## CASE PRESENTATION

**Figure 1:**
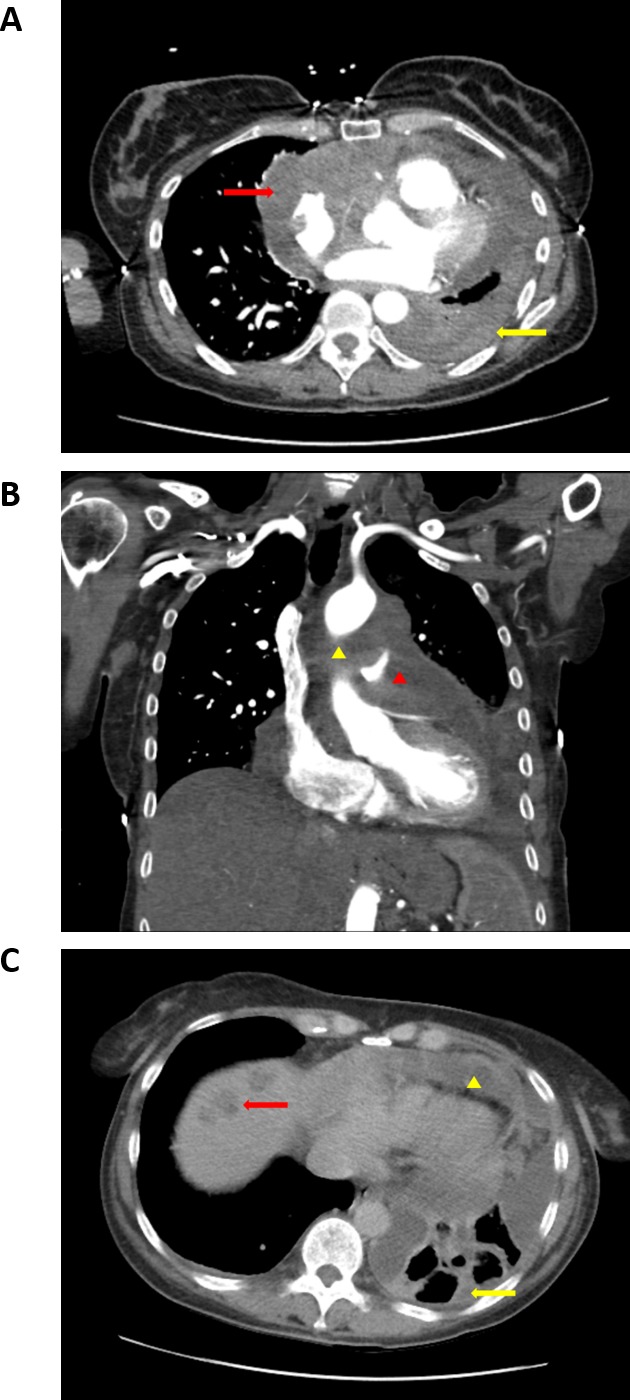
CT imaging of cardiac angiosarcoma prior to propranolol treatment **(A)** Baseline axial chest CT showing a large soft-tissue mass invading the right atrium and encasing the heart (red arrow). There is also nodular pleural thickening (yellow arrow) along the costal and mediastinal pleural surfaces and a small pleural effusion secondary to pleural infiltration. **(B)** Baseline coronal chest CT shows soft tissue mass encasing the ascending aorta (yellow arrowhead) and main pulmonary artery (yellow arrowhead). **(C)** Baseline axial chest CT shows circumferential pericardial thickening and effusion (yellow arrowhead) and nodular pleural thickening (yellow arrow) with loculated pleural effusion. Note small hypodense lesions in the right lobe of the liver consistent with metastases (red arrow).

In late 2016, a 61 year old non-smoking female presented with exertional shortness of breath, mild pedal edema, distended neck veins, and a recent weight gain of 15 lbs over the prior 2 weeks. It was initially suspected that the patient was experiencing pulmonary embolism based on her clinical presentation and elevated D-dimer, but further testing revealed that she was experiencing cardiac tamponade with mild pulmonary hypertension and heart failure. Pericardiocentesis was performed without definitive diagnosis. In February 2017, the patient exhibited worsening respiratory symptoms without fever, hemoptysis, sputum production, B symptoms, or extremity edema. Baseline chest PET-CT images were reviewed by radiologists at both UCLA Oncology and Texas Tech University Health Sciences Center, demonstrating a large middle mediastinal mass encasing the main pulmonary artery, with pericardial and left pleural effusion (Figure 1A & Figure 1B). Scattered pulmonary nodules and hypodense lesions were identified in the right lobe of the liver, consistent with a diagnosis of metastatic disease (Figure 1C). Biopsy of the mediastinal mass revealed a high grade undifferentiated malignant neoplasm composed of highly proliferative (ki67 staining ~50%) pleomorphic anaplastic epithelioid malignant cells with large areas of necrosis and fibrosis. Immunohistochemistry revealed strong antigenicity for CD31 and CD34, and weak antigenicity for D2-40 and Factor VIII indicating a diagnosis of angiosarcoma that was corroborated at both UC San Diego Health and MD Anderson.

**Figure 2:**
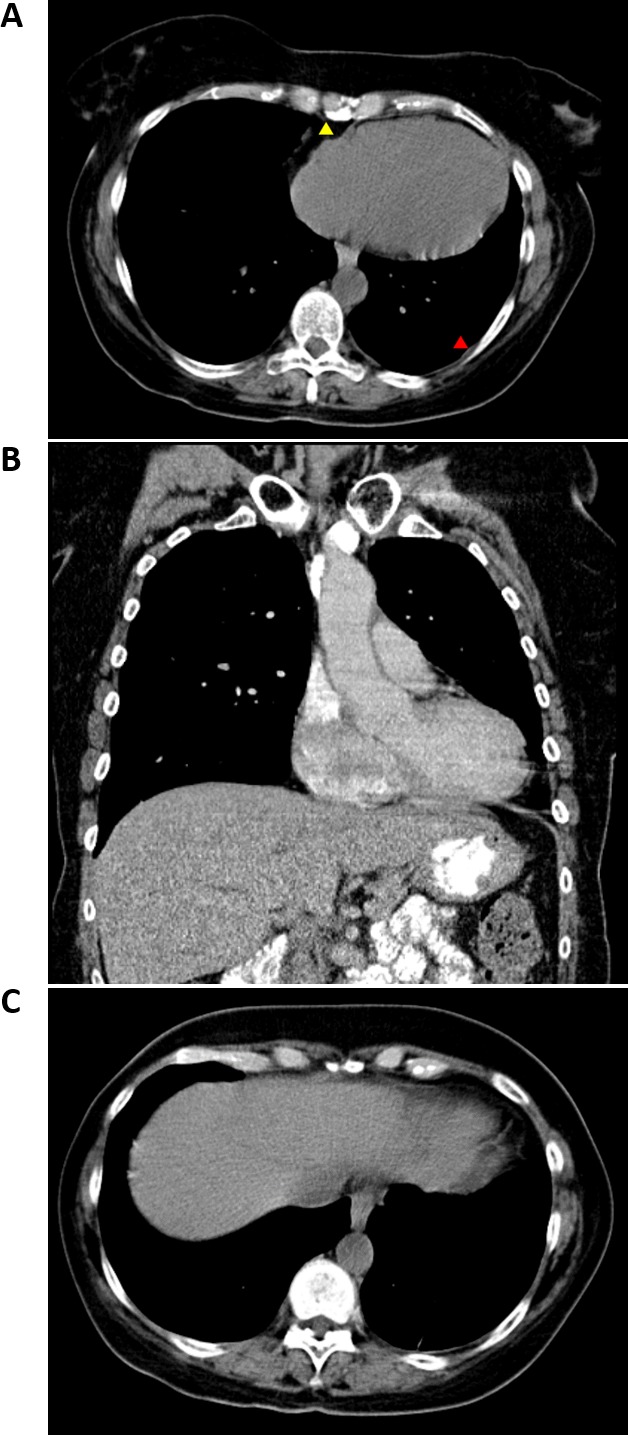
CT imaging of cardiac angiosarcoma subsequent to propranolol treatment **(A)** Axial chest CT following treatment with non-selective beta blocker shows marked reduction in size of the pericardial and pleural mass. There is minimal residual pleural (red arrowhead) and pericardial thickening (yellow arrowhead). **(B)** Coronal chest CT shows regression of soft tissue mass encasing the ascending aorta and main pulmonary artery after treatment with non-selective beta blocker. **(C)** Axial CT abdomen following treatment with non-selective beta blocker shows resolution of liver metastases.

Conventional treatment options were recommended, however the patient declined these based on low reported survival rates, and instead, requested the non-selective beta blocker propranolol as a single agent therapy. In May 2017, 40 mg/kg propranolol was administered daily and PET-CT scans were performed at regular intervals to assess the response of the tumor to propranolol. Assessment of tumor response was based on 18F-fluorodeoxyglucose (FDG) tracer uptake and measurements/assessments of the primary tumor and distant metastases. After 12 months of propranolol as a single agent therapy, significant debulking and decreased size of the residual mediastinal mass was observed on PET-CT scans, with resolution of pericardial effusion (Figure 2A & Figure 2B). Pulmonary nodules were stable to regressed, and the nodules in the right lobe of the liver had completely resolved (Figure 2C). There was no evidence of residual hyper-metabolic activity based on FDG measurements in the primary lesion or in metastatic sites in the chest, abdomen, or pelvis on PET-CT.

## DISCUSSION

The targets of propranolol, the beta adrenergic receptor proteins (β-AR1, β-AR2, β-AR3), are expressed in angiosarcoma [25, 27]. Preclinical studies using *in vitro* and *in vivo* angiosarcoma models indicate that propranolol as a single agent is capable of reducing tumor cell proliferation, selectively inducing tumor cell apoptosis while sparing non-diseased cell types, and inhibiting the growth of angiosarcoma xenografts in animal tumor models [23, 25, 26]. Translation of these findings into human patients revealed that propranolol alone is capable of reducing the angiosarcoma proliferative index after only 1 week of treatment [20], however the long term efficacy of propranolol as a single agent therapy was not individually evaluated in the aforementioned study, as the tumor was subsequently treated with radiation and chemotherapy. Non-randomized prospective and retrospective studies have combined propranolol with conventional anti-sarcoma therapies, leading to substantially increased progression free- and overall-survival of angiosarcoma patients [23, 26], however randomized clinical studies are necessary to comprehensively evaluate the efficacy and optimal dosing of propranolol against angiosarcoma. Several targeted therapeutics such as Pazopanib, TRC105, and others are currently being tested clinically against this tumor type. Future studies should evaluate which specific combination of conventional or targeted therapeutics maximizes patient survival when combined with propranolol.

Strong efficacy of propranolol as a single agent against other vascular tumor types has been reported for infantile hemangioma [28, 29], Kaposi's sarcoma [30], hemangioendothelioma [31-33], and hemangioblastoma [34-36]. Regardless of convention therapy, almost all patients will succumb to cardiac angiosarcoma within a year of diagnosis [14-18], and for patients who cannot or choose not to undergo surgery, chemotherapy, and/ or radiation, supportive care has historically remained their only remaining option. The current data presented in this report suggests that propranolol may have anti-tumor efficacy against cardiac angiosarcomas. Use of propranolol may be a viable alternative for patients who elect to forego conventional therapy.
