# Correction: Regression of primary cardiac angiosarcoma and metastatic nodules following propranolol as a single agent treatment

**DOI:** 10.18632/oncoscience.491

**Published:** 2019-09-08

**Authors:** Dana C. Galván, Anoop P. Ayyappan, Brad A. Bryan

**Affiliations:** ^1^ Paul L. Foster School of Medicine, Texas Tech University Health Sciences Center, El Paso, TX, USA; ^2^ Department of Radiology, Texas Tech University Health Sciences Center, El Paso, TX, USA; ^3^ Department of Biomedical Sciences, Texas Tech University Health Sciences Center, El Paso, TX, USA

**This article has been corrected:** A concentration in the Case Presentation section was corrected. The proper sentence is shown below.

## CASE PRESENTATION

…“In May 2017, 40 mg propranolol was administered daily and PET-CT scans were performed at regular intervals to assess the response of the tumor to propranolol.”

**Original article:** Oncoscience. 2018 Oct 11;5(9-10):264-8

***PMCID:*** PMC6231448 ***PMID:*** 30460329

***DOI:*** *10.18632/oncoscience.472*

